# (+)-Aeroplysinin-1 Modulates the Redox Balance of Endothelial Cells

**DOI:** 10.3390/md16090316

**Published:** 2018-09-06

**Authors:** Javier A. García-Vilas, Beatriz Martínez-Poveda, Ana R. Quesada, Miguel Ángel Medina

**Affiliations:** 1 Departamento de Biología Molecular y Bioquímica, Facultad de Ciencias, and IBIMA (Biomedical Research Institute of Málaga), Universidad de Málaga, Andalucía Tech, E-29071 Málaga, Spain; jandrovil@hotmail.com (J.A.G.-V.); bmpoveda@uma.es (B.M.-P.); quesada@uma.es (A.R.Q.); 2 CIBER de Enfermedades Raras (CIBERER), E-29071 Málaga, Spain

**Keywords:** aeroplysinin-1, angiogenesis, *Aplysina aerophoba*, endothelial cell, redox

## Abstract

The bioactive natural compound from marine origin, (+)-aeroplysinin-1, has been shown to exhibit potent anti-inflammatory and anti-angiogenic effects**.** The aim of the present study was to identify new targets for (+)-aeroplysinin-1 in endothelial cells. The sequential use of 2D-electrophoresis and MALDI-TOF-TOF/MS allowed us to identify several differentially expressed proteins. Four of these proteins were involved in redox processes and were validated by Western blot. The effects of (+)-aeroplysinin-1 were further studied by testing the effects of the treatment with this compound on the activity of several anti- and pro-oxidant enzymes, as well as on transcription factors involved in redox homeostasis. Finally, changes in the levels of total reactive oxygen species and mitochondrial membrane potential induced by endothelial cell treatments with (+)-aeroplysinin-1 were also determined. Taken altogether, these findings show that (+)-aeroplysinin-1 has multiple targets involved in endothelial cell redox regulation.

## 1. Introduction

Aeroplysinin-1 (Apl-1) is a chiral, optically active brominated compound produced by sponges of the order Verongida that has been described as a multi-targeted bioactive marine drug [1]. The best characterized isomer is the dextrorotatory (+)-Apl-1 enantiomer (Figure S1 in Supplementary Material), first isolated in 1972 [2]. The most stable conformer of (+)-Apl-1 is 2-[(1*S*,6*R*)-3,5-dibromo-1,6-dihydroxy-4-methoxycyclohexa-2,4-dien-1-yl]-acetonitrile. Structurally, (+)-Apl-1 presents a rigid skeleton due to the *cis* diene within the cyclohadiene ring and four flexible groups that are thought to be the interaction regions with its biological targets [3]. Initially identified as an antibacterial compound [2], (+)-Apl-1 was later shown to have a broad spectrum of antibiotic action against yeasts, dinoflagellates and retroviruses, among others [1]. Furthermore, it has also been shown that (+)-Apl-1 has either cytostatic or cytotoxic effects on several kinds of tumor, endothelial and monocyte cell lines [4,5,6,7,8]. Our group has been actively involved in the elucidation of some of the biological effects of (+)-Apl-1 described so far [1]. Years ago, our group published a complete study demonstrating for the first time that (+)-Apl-1 is a potent inhibitor of in vivo angiogenesis targeting multiple steps of the angiogenic process, as shown by using specific in vitro assays [6]. Years later, we demonstrated that the anti-angiogenic effect of (+)-Apl-1 is related to its apoptogenic effects on proliferative endothelial cells in culture [7]. Moreover, we have recently shown that (+)-Apl-1 inhibits both Akt and Erk phosphorylation in endothelial cells [9]. On the other hand, our group has also shown that (+)-Apl-1 can behave as an anti-inflammatory compound able to decrease the expression levels of cyclooxygenase-2 (COX-2) and monocyte chemoattractant protein-1 (MCP-1) in both endothelial and monocyte cell cultures [8]. Aiming to identify new potential targets for this bioactive compound, in the present study we carried out a proteomic approach based on the comparison of the spot patterns revealed by 2D electrophoresis of samples coming from (+)-Apl-1-treated and untreated RF-24 immortalized human umbilical vein endothelial cells and the identification of the differentially expressed spots by MALDI-TOF-TOF/MS. In fact, several redox proteins were affected by the treatment. Since compounds able to modulate the redox state have been proposed as promising for the therapy of angiogenesis-related diseases [10], the effects of (+)-Apl-1 on endothelial cell redox balance were further investigated. These and additional data here presented and discussed reveal that (+)-Apl-1 has remarkable modulatory effects on the redox balance of endothelial cells and shed new light on the previously described anti-angiogenic effect of this compound.

## 2. Results

### 2.1. (+)-Aeroplysinin-1 Affects the Expression Levels of Redox Proteins in RF-24 Endothelial Cells

**Figure 1:**
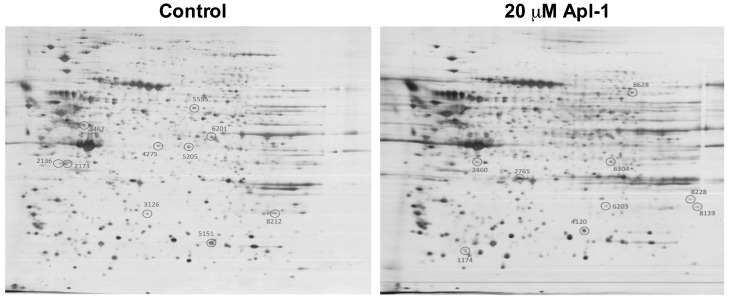
Differential expression of RF-24 cell proteins after 12 h of incubation in the absence (control) or presence of 20 µM (+)-Apl-1 as revealed by 2D electrophoresis. The whole procedure was carried out as described in the Materials and Methods section. Only those spots differentially expressed in a consistent way in three independent experiments (listed in Table 1 and Table 2) are circled in the representative 2D gel photographs.

To test the effects of (+)-Apl-1 on RF-24 endothelial cell proteome, the 2D electrophoresis of samples corresponding to the untreated and the 20 µM Apl-1 treated (for 12 h) RF-24 cells was performed. Figure S1 (in Supplementary Material) shows that 12 h of incubation in the presence of 20 μM Apl-1 had no cytotoxic effect on RF-24 cells. Figure 1 shows representative results of 2D electrophoresis highlighting differentially expressed spots.

**Figure 2:**
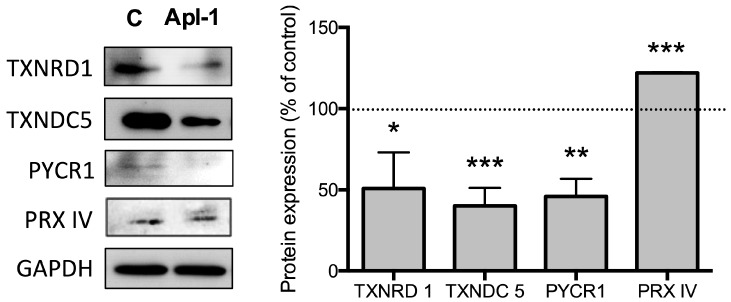
Western blot analysis of redox proteins differentially expressed. Three independent experiments were carried out. Representative images are shown. Quantification of bands is shown as relative values taking as 100% the intensity of bands corresponding to control, untreated cells. Data are given as means ± SD of three independent experiments. Significant differences between control-untreated and treated cells: *, *p* < 0.05; **, *p* < 0.01; ***, *p* < 0.005.

Spots were submitted to MALDI-TOF-TOF/MS for their identification. The identified proteins are involved in signal transduction pathways, glucose and redox metabolism (Table 1 and Table 2). We next confirmed the effects of (+)-Apl-1 on 4 redox proteins (thioredoxin reductase 1, TXNRD1; thioredoxin domain containing 5, TXNDC5; pyrroline-5-carboxylate reductase 1, PYCR1; and peroxiredoxin IV, PRX IV) by Western blotting (Figure 2).

### 2.2. (+)-Aeroplysinin-1 Affects Redox Enzyme Activities in RF-24 Endothelial Cells

**Figure 3:**
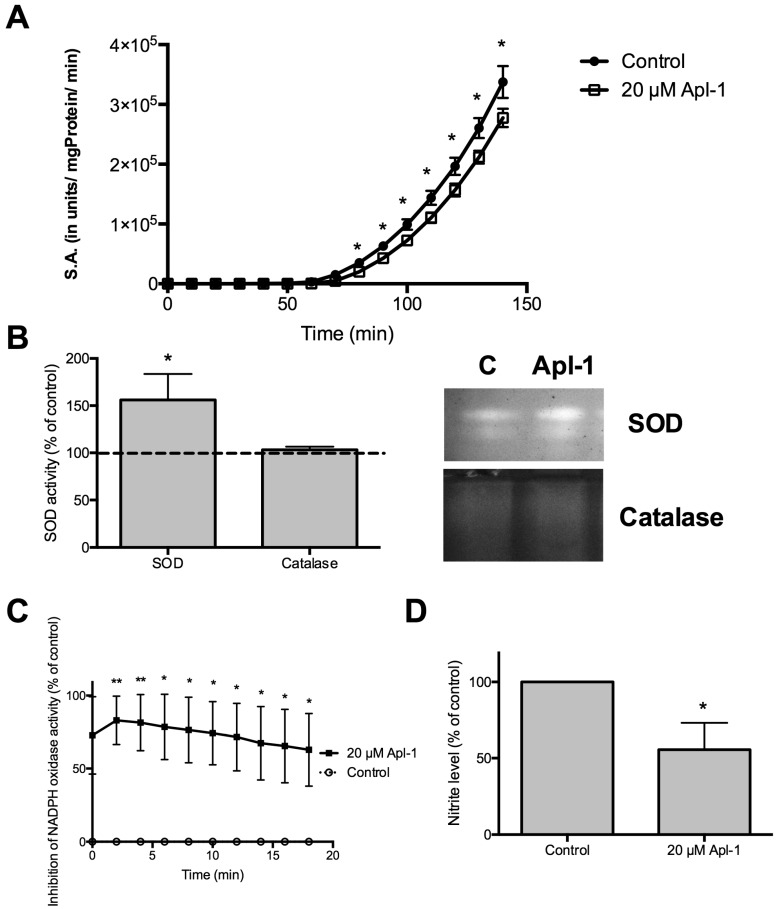
Effects of (+)-Apl-1 on redox enzymatic activity. (**A**) Protein disulphide isomerase (PDI) kinetics. PDI activity was determined as described in Materials and Methods. (**B**) Quantification of SOD and catalase activities in zymographies (carried out as described in Materials and Methods), taking control values of untreated cells as 100% and its quantification to control (**C**) and (+)-Apl-1 samples. Representative images of SOD and catalase zymographies. (**C**) Inhibition caused by (+)-Apl-1 on NADPH oxidase activity of RF-24 cells. (**D**) Quantification of nitrite with the Griess reagent as described in Materials and Methods. Data are given as means ± SD of three independent experiments. Significant differences between control-untreated and treated cells: * *p* < 0.05, ** *p* < 0.01.

Next, we focused our attention on the effects of (+)-Apl-1 on RF-24 cell redox systems. The enzymatic cellular redox system consists of a set of enzymes that generate ROS and reactive nitrogen species (RNS) and another set of antioxidant enzymes. TXNDC5 is an enzyme with protein disulphide isomerase (PDI) activity that can repair or correct protein conformation between reduced and oxidized thiol groups in oxidized proteins to prevent cell damage [11]. PRX IV can oxidize the thiol groups of PDI. Figure 3A shows that (+)-Apl-1 slightly decreased PDI activity of RF-24 cells. Zymographic assays of superoxide dismutase (SOD) and catalase activities revealed that Apl-1 treatment caused a significant increase of SOD activity but had no relevant effect on catalase (Figure 3B). On the other hand, (+)-Apl-1 strongly inhibited the activity of the pro-oxidant enzyme nicotinamide adenine dinucleotide phosphate (NADPH) oxidase (Figure 3C) and decreased the production of nitric oxide (NO) by nitric oxide synthase (NOS) (Figure 3D).

### 2.3. (+)-Aeroplysinin-1 Modulates Transcription Factors Involved in Redox Homeostasis

**Figure 4:**
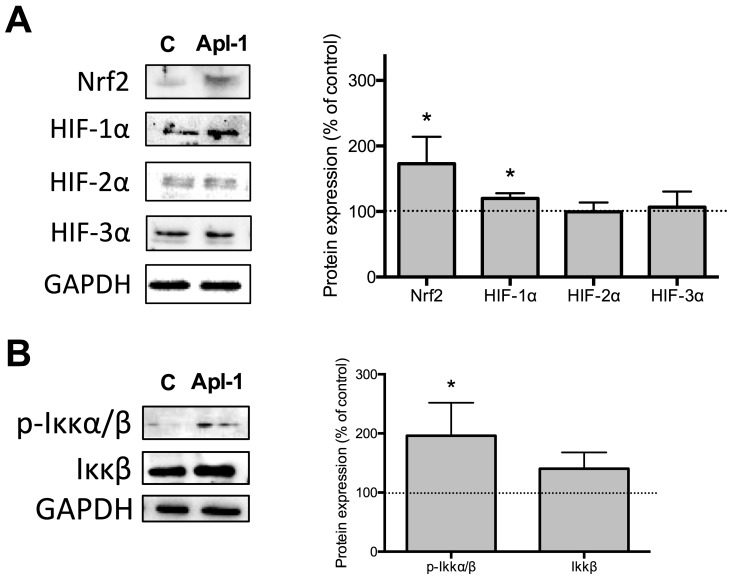
The effects of (+)-Apl-1 on redox regulator proteins. (**A**) Representative images showing the levels of Nrf2 and three HIF proteins in RF-24 cells after 12 h of incubation in the absence (control) or presence of 20 µM (+)-Apl-1 as revealed by Western blotting. Glyceraldehyde 3-phosphate dehydrogenase (GAPDH) bands are shown as internal controls. Quantification of bands is shown as relative values taking as 100% the intensity of bands corresponding to control, untreated cells. (**B**) Representative images showing the levels of Ikkβ and p-Ikkα/β in RF-24 cells after 12 h of incubation in the absence (control) or presence of 20 µM (+)-Apl-1 as revealed by Western blotting. GAPDH bands are shown as internal controls. Quantification of bands is shown as relative values taking as 100% the intensity of bands corresponding to the control, untreated cells. Data are given as means ± SD of three independent experiments. Significant differences between control-untreated and treated cells: *, *p* < 0.05.

The effects of (+)-Apl-1 on RF-24 endothelial cell transcription factors involved in the control of redox metabolism, such as hypoxia inducible factor 1α (HIF-1α), hypoxia inducible factor 2α (HIF-2α), hypoxia inducible factor 3α (HIF-3α), nuclear factor E2-related factor 2 (Nrf2), and nuclear factor kappa B (NF-κB) were also analyzed. Figure 4A shows the (+)-Apl-1 treatment increased the levels of Nrf2 and HIF-1α proteins in RF-24 cells. On the other hand, activation of NF-κB is controlled by Ikkβ, which avoids its nuclear translocation. However, when Ikkβ is phosphorylated (p-Ikkβ) it releases NF-κB. Figure 4B shows that 20 μM (+)-Apl-1 treatment for 12 h increased the levels of p-Ikkβ in RF-24 cells.

### 2.4. (+)-Aeroplysinin-1 Decreases ROS Levels and Mitochondrial Membrane Potential in RF-24 Endothelial Cells

**Figure 5:**
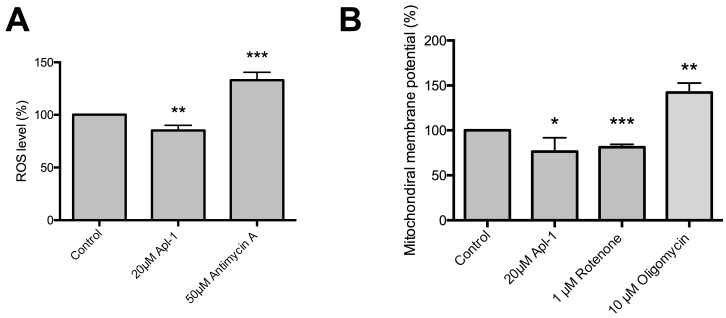
The effects of (+)-Apl-1 on reactive ROS levels and mitochondrial membrane potential. (**A**) Quantitative analysis of ROS level in control and (+)-Apl-1 samples. Antimycin A is a positive control to increase ROS level. (**B**) Quantitative analysis of mitochondrial membrane potential. Rotenone is a complex I inhibitor of the electron transport chain, and oligomycin is an adenosine triphosphate (ATP) synthase inhibitor. Data are given as means ± SD of three independent experiments. Significant differences between control-untreated and treated cells: *, *p* < 0.05; **, *p* < 0.01; ***, *p* < 0.005.

Figure 5A shows that an overall effect of 20 μM (+)-Apl-1 on RF-24 cell redox homeostasis is a decrease in the levels of ROS.

The measurement of the mitochondrial membrane potential (Δψ_m_) is an indirect way to test the mitochondrial functionality and its capacity to regulate both ATP and ROS production [12,13]. Figure 5B shows that 20 μM (+)-Apl-1 treatment slightly decreased RF-24 cell Δψ_m_ in a similar way to the inhibitory effect caused by the complex I inhibitor rotenone.

## 3. Discussion

In the present study, a proteomic approach based on the use of 2D electrophoresis, followed by spot identification by MALDI-TOF-TOF/MS, led us to identify the modulatory effects of (+)-Apl-1 on four redox proteins expressed by RF-24 human endothelial cells: PRX IV, TXNDC5, the cytoplasmic TXNRD1 and the mitochondrial PYCR1. We confirmed the effects of (+)-Apl-1 on these redox proteins by Western blotting. PRX IV belongs to the family of the peroxiredoxins, major scavengers of hydrogen peroxide, playing essential roles in redox signalling and oxidative stress. PRX IV is the only member of the peroxiredoxins located in the endoplasmic reticulum and has also a role in oxidative protein folding via oxidizing PDI [14]. Furthermore, PRX IV has a regulatory role in the activation of the transcription factor NF-κB. An extracellular PRX IV is bound to endothelial cells and is released to blood in response to variations in the redox conditions [15]. TXNDC5 is a PDI, a family of oxidoreductases involved in the oxidative folding of newly synthesized membrane proteins and secretory proteins. TXNDC5 is mainly expressed in liver and endothelial cells, although increased TXNDC5 levels have been reported in diseases in which oxygen is limited, including different types of cancer [16]. Moreover, TXNDC5 has been proposed as a biomarker and therapeutic target for several diseases. The contrasting results we observed for TXNDC5 and PRX IV agree with the overall light inhibitory effect of (+)-Apl-1 on PDI activity. TXNRD1 reduces oxidized cysteine residues of proteins and is required for the DNA binding of redox-sensitive transcription factors [17]. PYCR1 is anchored to the external mitochondrial membrane and catalyses the NAD(P)-dependent conversion of Δ^1^-pyrroline-5-carboxylate (P5C) to proline and ornithine. Furthermore, PYCR1 exerts protection against oxidative stress [18] and both P5C and proline have properties related to the cellular redox balance [19].

Our proteomic results confirmed by Western blot pointed to a previously undescribed role of (+)-Apl-1 in the modulation of endothelial cell redox homeostasis. An appropriate balance between the ROS-generating and ROS-scavenging enzymes is required for a correct cellular function, and their imbalance has been related with several diseases. Cells have specialized enzymes against different ROS. SODs detoxify the highly reactive superoxide anions (O_2_^−^), able to oxidize a broad spectrum of biomolecules [20]. A specific zymographic assay shows that (+)-Apl-1 was able to increase RF-24 endothelial cell SOD activity. Hydrogen peroxide (H_2_O_2_) is a non-radical ROS with toxic effects on cells. Cells fight against them with catalase, glutathione peroxidase and peroxidase enzymes. The catalase zymography results showed that (+)-Apl-1 did not have an effect on catalase activity. On the other hand, cells also have enzymes to generate ROS, such as nicotinamide adenine dinucleotide phosphate (NAPDH) oxidase, which consumes oxygen and produces O_2_^−^. This enzyme was discovered in neutrophils, but it is also present in endothelial cells, neurons, and astrocytes [21], with roles in normal physiology. However, its dysregulation is associated with numerous diseases. Since this enzyme is closely related to angiogenesis-induced diseases and we previously reported that (+)-Apl-1 is a potent anti-angiogenic compound [6], it seemed interesting to determine whether (+)-Apl-1 was able to affect NAPDH oxidase activity. Our results clearly show that, indeed, (+)-Apl-1 has an extremely potent inhibitory effect on NADPH oxidase activity. Nitric oxide synthase (NOS) produces nitric oxide (NO), which is an important molecule for normal physiological conditions [22]. However, excessive NO production can produce cytoxicity because it is a RNS able to affect protein function by nitrosylation [23,24]. Our results with the spectrophotometric assay based on the Griess reaction revealed that (+)-Apl-1 decreased the production of NO by RF-24 human endothelial cells.

The effects of (+)-Apl-1 on RF-24 human endothelial cell redox proteins described so far in the present study suggest a possible modulation of (+)-Apl-1 on the main transcription factors traditionally related with cellular redox stress, which are involved in redox-regulated endothelial cell fate. The key transcription factors involved in the redox regulation of endothelial cells are HIFs, Nrf-2 and NF-κB. The HIF family is composed of α and β subunits, with three different isoforms of α subunits (HIF-1α, HIF-2α, and HIF-3α). Both HIF-1α and HIF-2α are considered master sensors of hypoxia and are overexpressed in different types of cancer [25]. On the other hand, HIF-3α is less known, but there are distinct variants that appear to be involved in blockage of HIF-1α and HIF-2α [26]. Under hypoxic conditions, HIF-1α increases vascular endothelial growth factor (VEGF) and NOS, which are potent pro-angiogenic stimulators [27]. Nrf2 is a master transcription factor for many antioxidant and detoxifying enzyme genes [28]. Our results show increased HIF-1α and Nrf2 levels in RF-24 human endothelial cells treated with (+)-Apl-1, suggesting that (+)-Apl-1 could act as a protective agent against oxidative stress, thus preventing angiogenic effects induced by ROS. In our study, we included the analysis of Ikk, given that its modulation regulates NF-κB transcription factor activity. NF-κB regulates the expression of genes involved in apoptosis, inflammation, immune response, cell adhesion, proliferation, and cellular-stress response [29]. It has been reported that Ikkβ activity is dysregulated in inflammatory diseases and cancer [30,31], both closely related to angiogenesis. Ikkβ joins to NF-κB, thus avoiding NF-κB nuclear translocation. By contrast, Ikkβ phosphorylation leads to NF-κB release and its nuclear translocation. Ikk inhibition has been proposed as a potential anti-angiogenic and anti-tumor therapy [30,31]. The potent inactivation of Ikk by phosphorylation induced by (+)-Apl-1 in RF-24 human endothelial cells is a novel clue explaining how (+)-Apl-1 could exert its previously described anti-inflammatory and anti-angiogenic effects [6,8] at least in part by its modulation of the master inflammation and cell stress response NF-κB pathway.

All the results discussed so far suggest that (+)-Apl-1 can be described as a bioactive compound with multiple targets involved in endothelial cell redox homeostasis. Therefore, inhibitory effects of (+)-Apl-1 on global indicators of the cellular redox state such as ROS levels and changes in Δψ_m_ could be expected. Several reports have related ROS with angiogenesis-related diseases, such as Alzheimer’s disease, Parkinson’s disease, diabetes, cardiovascular diseases, hypertension and cancer, among others [32,33,34,35,36,37]. Therefore, proteins and cellular processes modulated by ROS have been proposed as new molecular targets to design novel anti-angiogenic therapies [35,38]. Our results with (+)-Apl-1 show that this compound was able to decrease RF-24 human endothelial cell ROS levels, thus exhibiting an ability to protect against oxidative stress similar to that previously described for epigallocatechin gallate (EGCG) [39], curcumin [40], and other antioxidants. On the other hand, Δψ_m_ is a parameter that indicates the oxidative energy metabolism, and it is also an indirect parameter to evaluate the proton motive force, since ATP production is directly associated with Δψ_m_ polarization [41]. Δψ_m_ dysregulation is associated with neurodegenerative diseases [42] and cancer [43]. For this reason, there is an increasing interest in the therapeutic potential of mitochondrial-directed antioxidants [44]. In the present work, we show that (+)-Apl-1 reduced endothelial cell Δψ_m_ as previously shown for prazosin, another anti-angiogenic compound [45].

Overall, our findings show that (+)-Apl-1 has multiple targets involved in the redox regulation of RF-24 human endothelial cells and shed new light on its previously described anti-inflammatory and anti-angiogenic effects [1,6,8,46]. In summary, the data presented here suggest a potential application of (+)-Apl-1 for the treatment of angiogenesis-dependent diseases. Future translational studies to evaluate this potential application of (+)-Apl-1 should be warranted.

## 4. Materials and Methods

### 4.1. Materials

(+)-Apl-1 was provided by Instituto Biomar (León, Spain) and was dissolved in DMSO and stored at −20 °C. Supplements and other chemicals not listed in this section were obtained from Sigma Chemicals Co. (St. Louis, MO, USA). Cell culture media, penicillin, streptomycin and amphotericin B were purchased from Biowhittaker (Walkersville, MD, USA). Fetal bovine serum (FBS) and human serum were products of Harlan-Seralb (Belton, UK). Antibodies (anti-TXNRD1, anti-TXNDC5, anti-PYCR1, and anti-PRX IV) were acquired from AbCam (Cambridge, UK). Antibodies (anti-GAPDH, anti-p-Iκκα/β, anti-Iκκβ) were purchased from Cell Signaling Technology (Danvers, MA, USA). Antibodies (anti-Nrf2) were purchased from Santa Cruz Biotechnologies (Dallas, TX, USA). Secondary antibodies (anti-rabbit IgG, horseradish peroxidase-linked whole antibody, and anti-mouse IgG, horseradish peroxidase-linked whole antibody) were acquired from GE Healthcare (Buckinghamshire, UK). Plastics for cell culture were supplied by NUNC (Roskilde, Denmark) and VWR (West Chester, PA, USA).

### 4.2. Cell Culture

Immortalized human umbilical vein endothelial cells (RF-24) were kindly supplied by Dr. Arjan W. Griffioen (Maastrich University, Maastrich, The Netherlands), who previously characterized them elsewhere [47]. This endothelial cell line was grown in RPMI-1640 medium supplemented with glutamine (2 mM), amphotericin B (1.25 mg/mL), penicillin (50 IU/mL), streptomycin (50 mg/L), 10% fetal bovine serum and 10% human serum. Cell cultures were maintained at 37 °C under a humidified 5% CO_2_ atmosphere.

### 4.3. MTT Cell Survival Assay

The 3-(4,5-dimethylthiazol-2-yl)-2-5-diphenyltetrazolium bromide (MTT) dye reduction assay in 96-well microplates was used. The assay is dependent on the reduction of MTT by mitochondrial dehydrogenases of viable cells to a blue formazan product, which can be measured spectrophotometrically. Endothelial cells (2.5 × 10^3^ cells in a total volume of 100 μL of complete medium) were incubated in each well with serial dilutions of (+)-Apl-1. After 12 h of incubation (37 °C, 5% CO_2_ in a humid atmosphere), 10 μL of MTT (5 mg/mL in PBS) was added to each well, and the plate was incubated for a further 4 h (37 °C). The formazan was dissolved in 150 μM of 0.04 N HCl-2 propanol, and samples were spectrophotometrically measured at 550 nm. All determinations were carried out in quadruplicate, and at least three independent experiments were carried out.

### 4.4. Sample Preparation for Proteomic Analysis, 2-D Electrophoresis and Protein Identification

Cells were grown to 80–90% confluence. Then, cells were washed twice with PBS, and incubated for 12 h at 37 °C in the absence or presence of aeroplysinin-1 (20 μM). After incubation, cells were washed three times with PBS, and were briefly trypsinized. Next, cells were harvested and centrifuged for 5 min at 1200× *g* at 4 °C. Subsequently, cells were lysed directly in 500 μL of 2-D lysis buffer (ethylenediaminetetraacetic acid (EDTA) 1 mM, urea 7 M, thiourea 2 M, 3-[(3-cholamidopropyl)dimethylammonio]-1-propanesulfonate hydrate (CHAPS) 4%, sodium dodecyl sulfate (SDS) 0.1%). Samples were clarified by centrifugation for 10 min at 15,000 g and were then applied to nonlinear pH 3–10 immobilized pH gradient strips. Next, isoelectric focusing was performed, and strips were then equilibrated for 15 min in SDS-equilibration buffer supplemented with 1% (*w*/*v*) DL-dithiothreitol (DTT) and for another 15 min with SDS-equilibration buffer supplemented with 2.5% (*w*/*v*) iodoacetamide. After equilibration, strips were applied to 10% sodium dodecyl sulfate-polyacrylamide gel electrophoresis (SDS–PAGE) gels. Electrophoresis was carried out at 2.5 W per gel during the first 30 min followed by 17 W per gel until complete. Proteins in the gels were fixed and stained using the silver staining procedure as described by Shevchenko et al. [48] and were stored at 4 °C in 2% (*v*/*v*) acetic acid until mass spectrometry (MS) analysis. For gel-image analysis, gels were scanned at high resolution with a calibrated densitometer model GS-800 (Bio-Rad, Hercules, CA, USA), and the PDQuest version 7.4 software (Bio-Rad) was used for detection of qualitative and quantitative alterations in protein spots. Spots of interest were excised from the gels, washed twice with water and were in-gel digested with porcine trypsin (Promega, Madison, WI, USA) in essence as described by Shevchenko et al. [47]. The generated peptides were analyzed by MS using a 4700 MALDI-TOF/TOF mass spectrometer (Applied Biosystems, Foster City, CA, USA). Protein identification was achieved by a combined strategy consisting of a peptide mass fingerprinting (PMF) search plus the MS/MS search of up to five peptide ions. Searches were performed using GPS Explorer™ software v 3.5 (Applied Biosystems) in non-redundant NCBI database of proteins using MASCOT searching engine (Matrix Science Ltd., London, UK). Digestion of proteins in the spots, MS and PMF searches were performed by the “Unidad de Proteómica” (Bioinnovation Building, University of Málaga, Málaga, Spain). The procedure was carried out in three independent experiments.

### 4.5. Western Blot

Cells were lysed with radioimmunoprecipitation assay (RIPA) buffer and the samples were quantified with Bradford protein assay. Samples were mixed with Laemmli’s loading buffer 6× and boiled for 5 min at 95 °C. Then, samples were separated by SDS-PAGE electrophoresis and blotted onto nitrocellulose membranes using standard procedures. After blocking in TBS-T plus 5% non-fat dry milk, membranes were probed with primary antibodies overnight at 4 °C. Then, membranes were washed in tris-buffered saline and polysorbate 20 (TBS-T) and probed with horseradish peroxidase (HPR)-conjugated secondary antibodies in a blocking solution buffer for 1 h at room temperature. After washing, membranes were developed using the enhanced chemiluminescence (ECL) system (Amersham Biosciences, Amersham, UK). For antibody re-probing, membranes were incubated in Restore Western Blot Stripping Buffer (Thermo Scientific, Waltham, MA, USA) for 15 min at 37 °C with shaking.

### 4.6. PDI Activity Assay

Cells treated or untreated with 20 μM Apl-1 for 12 h were lysed with 50 mM phosphate buffer pH 7.8 on ice for 30 s using a sonicator microtip probe at 40% power. Each assay was done mixing reaction solution (100 mM Na_2_HPO_4_, 100 mM Na-EDTA, 10 mg/mL insulin, 100 mM DTT) with 15 μg of sample. The mix was incubated for 5 min at 37 °C and the absorbance at 650 nm was measured every 10 min. Protein concentration was determined with the Bradford method [49].

### 4.7. SOD and Catalase Zymographies

Cells treated or untreated with 20 μM Apl-1 for 12 h, were lysed with 50 mM phosphate buffer pH 7.8 on ice for 30 s using sonicator microtip probe at 40% power. Protein concentration was determined with the Bradford method [49]. Samples were loaded in acrylamide/bis-acrylamide gels (8% for SOD activity, and 12% for catalase activity). To visualize SOD activity, gels were stained with 2.43 mM nitro blue tetrazolium chloride (NBT), 28 mM tetramethylethylenediamine (TEMED) and 0.14 M rivoflavin-5′-phosphate for 20 min. Gels were washed with _dd_H_2_O and light exposure for 5 min. To visualise catalase activity, acrylamide/bis-acrylamide gels were incubated with a 0.003% H_2_O_2_ solution for 10 min. Gels were stained with 2% (*w*/*v*) ferric chloride and 2% (*w*/*v*) potassium ferricyanide. In both cases, enzymatic activity was detected as achromatic bands.

### 4.8. Assay of NADPH Oxidase Activity

NADPH oxidase activity was assayed by cytochrome *c* reduction quantified at 540 nm in cultured cells. Cells were grown to 80–90% confluence in a 96-well plate. Before performing the NADPH oxidase activity assay, cells were washed twice with PBS and incubated for 12 h at 37 °C in the absence or presence of 20 μM Apl-1. Thereafter, cells were washed twice with PBS, and fresh culture medium supplemented with 200 μM NADPH and 60 μM cytochrome *c* was added to every well with cells. The 96-well plate was incubated for 5 min at 37 °C to equilibrate the cells with the fresh culture medium supplemented. The spectrophotometer was set up to 540 nm and run the kinetic program at 37 °C. The absorbance values were measured every 2 min for 20 min.

### 4.9. Spectrophotometric Measurement of Nitrite as an Indicator of Nitric Oxide Production

The quantity of nitrite in the culture medium was measured as an indicator of nitric oxide production. Nitrite was quantified using the Griess reagent (1% sulfanilamide and 0.1% naphthylethylenediamine dihydrochloride in 5% phosphoric acid). 50 μL of cell culture medium was mixed with 50 μL of Griess reagent, and the mix was incubated at room temperature in the dark for 15 min. Absorbance at 540 nm was measured. The quantity of nitrite in samples was determined by interpolation in a sodium nitrite standard curve.

### 4.10. Measurement of Intracellular ROS Levels

Cells grown to 80–90% confluence in 6-well plates were washed twice with PBS. Treatment was carried out in serum free culture medium for 12 h. Once removed the medium, cells were loaded with 10 μM 2,7-dicholorodihydrofluorescein diacetate (DCFH-DA) at 37 °C for 20 min in the dark. Then, cells were washed in PBS and samples were analysed by using a FACSVerse flow cytometer (BD Biosciences, Franklin Lakes, NJ, USA).

### 4.11. Measurement of Intracellular Mitochondrial Membrane Potential

After 12 h of treatment, cells were harvested, washed with PBS and loaded with Rhodamine 123 at 37 °C for 30 min in the dark. Then, cells were washed with PBS and samples were analysed by using a FACSVerse flow cytometer (BD Biosciences).

### 4.12. Statistical Analysis

For all of the assays, at least three independent experiments were carried out. Results are expressed as the mean ± SD. Statistical significance was determined by using a Student’s paired simple test. Values of *p* < 0.05 were considered to be significant.

## Figures and Tables

**Table 1:** List of proteins whose levels were decreased with 20 μM (+)-Apl-1 treatment.

ID Gel	Protein	Gen	Function
2136	Ubiquitin-conjugating enzyme E2 1	UBC1	Catalyzes the covalent attachment of ubiquitin to other proteins. Functions in degradation of misfolded or regulated proteins localized in the endoplasmic reticulum (ER) lumen or membrane via the ubiquitin-proteasome system.
2173	Lactoylglutathione lyase	GLO1	Catalyzes the conversion of hemimercaptal, formed from methylglyoxal and glutathione, to S-lactoylglutathione.
3126	Chloride intracellular channel protein 4	CLIC4	Can insert into membranes and form poorly selective ion channels that may also transport chloride ions. Has alternate cellular functions like a potential role in angiogenesis or in maintaining apical-basolateral membrane polarity during mitosis and cytokinesis. Could also promote endothelial cell proliferation and regulate endothelial morphogenesis (tubulogenesis).
3467	TXNDC5 protein	TXNDC5	Cell redox homeostasis. TXNCD5 is a protein-disulfide isomerase. Its expression is induced by hypoxia and its role may be to protect hypoxic cells from apoptosis.
4275	L-lactate dehydrogenase B chain	LDHB	Catalytic activity (S)-lactate + NAD+ = pyruvate + NADH
5151	Triosephosphate isomerase	TPI1	d-glyceraldehyde 3-phosphate = glycerone phosphate.
5205	Transaldolase	TALDO1	Transaldolase is important for the balance of metabolites in the pentose-phosphate pathway. Sedoheptulose 7-phosphate + d-glyceraldehyde 3-phosphate = d-erythrose 4-phosphate + d-fructose 6-phosphate.
5555	Thioredoxin reductase 1, cytoplasmic	TXNRD1	Isoform 1 may possess glutaredoxin activity as well as thioredoxin reductase activity and induces actin and tubulin polymerization, leading to formation of cell membrane protrusions. Isoform enhances the transcriptional activity of estrogen receptors alpha and beta while isoform enhances the transcriptional activity of the beta receptor only.
6201	Annexin A1	ANXA1	Calcium/phospholipid-binding protein which promotes membrane fusion and is involved in exocytosis. This protein regulates phospholipase A2 activity.
8212	Pyrroline-5-carboxylate reductase 1, mitocondrial	PYCR1	Housekeeping enzyme that catalyzes the last step in proline biosynthesis. Can utilize both NAD and NADP, but has higher affinity for NAD. Involved in the cellular response to oxidative stress. l-proline + NAD(P)+ = 1-pyrroline-5-carboxylate + NAD(P)H.

**Table 2:** List of proteins whose levels were increased with 20 μM (+)-Apl-1 treatment.

ID Gel	Protein	Gen	Function
1174	Vimentin	VIM	Vimentins are class-III intermediate filaments found in various non-epithelial cells, especially mesenchymal cells.
2460	Keratin, type II cytoskeletal 7	KRT7 SCL	Blocks interferon-dependent interphase and stimulates DNA synthesis in cells. Involved in the translational regulation of the human papillomavirus type 16 E7 mRNA (HPV16 E7).
2765	Heat shock protein HSP 90-beta	HSP90ß	Molecular chaperone that promotes the maturation, structural maintenance, and proper regulation of specific target proteins involved for instance in cell cycle control and signal transduction.
4120	Peroxiredoxin-4	PRDX4	Probably involved in redox regulation of the cell. Regulates the activation of NF-kappa-B in the cytosol by a modulation of I-kappa-B-alpha phosphorylation.
6203	Estrogen sulfotransferase	SULT1E1 STE	May control the level of the estrogen receptor by sulfurylating free estradiol. 3′-phosphoadenylyl sulfate + estrone = adenosine 3′,5′-bisphosphate + estrone 3-sulfate.
6304	GDP-L-fucose synthase	TSTA3 SDR4E1	GDP-l-fucose + NADP+ = GDP-4-dehydro-6-deoxy-d-mannose + NADPH.
8139	Guanine nucleotide-binding protein subunit beta-2-like 1	GNB2L1 HLC7 PIG21	Involved in PKC-dependent translocation of ADAM12 to the cell membrane. Promotes the ubiquitination and proteasome-mediated degradation of proteins such as CLEC1B and HIF1A.
8228	Voltage-dependent anion-selective channel protein 2	VDAC2	Forms a channel through the mitochondrial outer membrane that allows diffusion of small hydrophilic molecules. The channel adopts an open conformation at low or zero membrane potential and a closed conformation at potentials above 30–40 mV. The open state has a weak anion selectivity whereas the closed state is cation-selective.
8628	Transketolase	TKT	Sedoheptulose 7-phosphate + d-glyceraldehyde 3-phosphate = d-ribose 5-phosphate + d-xylulose 5-phosphate.

## Supplementary Materials

The following are available online at http://www.mdpi.com/1660-3397/16/9/316/s1, a pdf file containing the Figure S1 legend and Figure S1.
